# Nature and extent of person recognition impairments associated with Capgras syndrome in Lewy body dementia

**DOI:** 10.3389/fnhum.2014.00726

**Published:** 2014-09-24

**Authors:** Chris M. Fiacconi, Victoria Barkley, Elizabeth C. Finger, Nicole Carson, Devin Duke, R. Shayna Rosenbaum, Asaf Gilboa, Stefan Köhler

**Affiliations:** ^1^Department of Psychology, The Brain and Mind Institute, University of Western OntarioLondon, ON, Canada; ^2^Department of Psychology, York UniversityToronto, ON, Canada; ^3^Department of Clinical Neurological Sciences, Schulich School of Medicine, University of Western Ontario, LondonON, Canada; ^4^Rotman Research Institute, BaycrestToronto, ON, Canada; ^5^Department of Psychology, University of TorontoToronto, ON, Canada

**Keywords:** Capgras syndrome, Lewy body dementia, interoceptive awareness, person recognition, affect perception

## Abstract

Patients with Capgras syndrome (CS) adopt the delusional belief that persons well-known to them have been replaced by an imposter. Several current theoretical models of CS attribute such misidentification problems to deficits in covert recognition processes related to the generation of appropriate affective autonomic signals. These models assume intact overt recognition processes for the imposter and, more broadly, for other individuals. As such, it has been suggested that CS could reflect the “mirror-image” of prosopagnosia. The purpose of the current study was to determine whether overt person recognition abilities are indeed always spared in CS. Furthermore, we examined whether CS might be associated with any impairments in overt affective judgments of facial expressions. We pursued these goals by studying a patient with Dementia with Lewy bodies (DLB) who showed clear signs of CS, and by comparing him to another patient with DLB who did not experience CS, as well as to a group of healthy control participants. Clinical magnetic resonance imaging scans revealed medial prefrontal cortex (mPFC) atrophy that appeared to be uniquely associated with the presence CS. We assessed overt person recognition with three fame recognition tasks, using faces, voices, and names as cues. We also included measures of confidence and probed pertinent semantic knowledge. In addition, participants rated the intensity of fearful facial expressions. We found that CS was associated with overt person recognition deficits when probed with faces and voices, but not with names. Critically, these deficits were not present in the DLB patient without CS. In addition, CS was associated with impairments in overt judgments of affect intensity. Taken together, our findings cast doubt on the traditional view that CS is the mirror-image of prosopagnosia and that it spares overt recognition abilities. These findings can still be accommodated by models of CS that emphasize deficits in autonomic responding, to the extent that the potential role of interoceptive awareness in overt judgments is taken into account.

## INTRODUCTION

Misidentification syndromes are among the most fascinating and puzzling forms of memory problems that can result from psychiatric or neurological disease. They are monothematic delusions that have intrigued psychologists and philosophers alike for over a century, but have only recently been brought into the realm of scientific investigation. Misidentification syndromes have been observed in relation to places, objects, and people, and have become known collectively as “delusions of misidentification.” Perhaps the most striking condition is Capgras syndrome (CS), in which individuals come to adopt the delusional belief that persons well-known to them have been replaced by an impostor or a “double.” A defining characteristic of delusions that is also present in CS is that patients will firmly hold on to their delusional beliefs in the presence of mounting contradictory evidence. While commonly observed in the context of psychiatric disease, CS can also result from various neurological conditions. The purpose of the current study is to shed more light on the nature of cognitive and affective deficits associated with CS in the context of neurodegenerative disease.

### CAPGRAS SYNDROME: A GENERAL FRAMEWORK FOR UNDERSTANDING FUNCTIONAL IMPAIRMENTS

Given its close association with psychiatric illness (e.g., paranoid schizophrenia and other psychoses), it was thought for many years that CS is the result of abnormal psychodynamic processes. However, research conducted over the past 40 years has attempted to ground CS symptomatology in brain-based dysfunction within face-processing and person recognition models (Ellis et al., 1997; Ellis and Lewis, 2001). In this newer endeavor, it has been hypothesized that patients can normally map faces onto stored representations of known individuals based on computations performed within structures of the ventral visual pathway (Bauer, 1984; Bauer and Verfaellie, 1988; Ellis and Young, 1990; Ellis et al., 1997; Ellis and Lewis, 2001). Critically, however, recognition processes computed within a second pathway, which diverges after initial structural analysis and allows for the generation of an appropriate affective signal to familiar individuals, is proposed to be impaired. This second pathway is thought to be comprised of limbic structures, such as the amygdala, and possibly frontal regions, including the insula and anterior cingulate cortex (Breen et al., 2000). Alternatively, it has been suggested that CS could result from a disconnection between ventral visual structures and limbic structures dedicated to affective processing. Evidence in favor of accounts that link CS to affective processing has come from a handful of case studies in patients with CS caused by a variety of etiologies, which revealed reduced autonomic responses [as measured by skin conductance responses (SCRs)] to pictures of personally known individuals and of famous people (Ellis et al., 1997; Hirstein and Ramachandran, 1997; Brighetti et al., 2007). Based on these findings, it has been hypothesized that the monothematic delusional belief that characterizes CS is a result of the patients’ attempt to “make sense” of the absence of an expected affective signal (e.g., Young, 2008).

In functional terms, the “cognitive” and “affective” routes to face recognition described above have been suggested to underlie overt and covert aspects of face-processing, respectively (Tranel and Damasio, 1985, 1988; Bauer and Verfaellie, 1988). Overt face recognition refers to the ability to make accurate explicit recognition judgments for faces, whereas covert face recognition refers to signs of an implicit differentiation between familiar and unfamiliar faces in behavior, or most pertinent in the context of CS, at the level of autonomic responding. Evidence in favor of this distinction has come from neuropsychological patient studies that revealed a double dissociation between these two forms of face recognition (Tranel et al., 1995). Specifically, bilateral lesions to ventro-medial prefrontal cortex (vmPFC) have been shown to disrupt covert face recognition, as assessed using SCR, while leaving overt face recognition intact. By contrast, bilateral lesions to occipito-temporal cortex resulted in impairments in overt but not covert face recognition judgments (see also Bauer, 1984; Tranel and Damasio, 1985; Bauer and Verfaellie, 1988, for similar findings in other prosopagnosic patients). The preservation of covert face recognition in prosopagnosic patients also stands in contrast to findings of impaired covert face recognition, as reflected in reduced SCR responses, in CS (Ellis et al., 1997; Hirstein and Ramachandran, 1997; Brighetti et al., 2007). Indeed, some researchers have endorsed the view that the face recognition deficits in CS are the mirror-image of those observed in prosopagnosia (Ellis and Lewis, 2001).

According to the two-factor framework of delusions, deficits in affective reactivity in face recognition may not provide a full account of CS (Coltheart, 2010; Coltheart et al., 2011). Coltheart (2010) has argued that such deficits in isolation cannot explain why CS patients typically remain firmly wedded to their delusional beliefs, even when strong counter-evidence (e.g., a wedding band with engraving) is presented. To explain these observations, Coltheart (2010) proposed that CS involves an additional deficit in executive control processes that are necessary for monitoring the contents of memory retrieval, as well as the critical evaluation of hypotheses. Evidence in support of this idea comes from observations that many cases of CS, and other delusions, have been found to be associated with right prefrontal cortical damage (Alexander et al., 1979; Staff et al., 1999; Corlett et al., 2007; Devinsky, 2009; Ismail et al., 2012; Thiel et al., 2014). Regardless of whether a second factor is required to explain CS (see Corlett et al., 2010, for critique), the two-factor model as well as previous single-factor models (e.g., Breen et al., 2000; Ellis and Lewis, 2001) emphasize the presence of a deficit in covert familiarity responses that leaves overt person recognition judgments intact. Here we examine this central notion in the literature on CS more closely.

### NATURE AND EXTENT OF PERSON RECOGNITION DEFICITS IN CAPGRAS SYNDROME

When thinking about the functional impairments that characterize CS, perhaps the most critical issue is the extent to which person recognition is affected. What appears puzzling, at least at first glance, is that the delusion appears to be restricted to one or a small number of individuals who typically have close emotional bonds to the patient. However, it would seem premature to conclude solely based on the scope of the delusion and anecdotal reports from relatives that abnormalities in person recognition are indeed restricted to loved ones. Rather, this issue requires systematic investigation of person recognition abilities with controlled experimental tasks.

Recent psychophysiological research in healthy individuals has demonstrated that exposure to faces of loved ones is associated with a larger autonomic response than exposure to other well-known people as reflected in SCR, heart rate, and other psychophysiological measures (Vico et al., 2010; Guerra et al., 2011, 2012). However, the generation of autonomic arousal is not unique to exposure to loved ones in person recognition. In fact, several studies have revealed increased autonomic arousal for famous people as compared to unfamiliar people as well (Tranel et al., 1985; Bauer and Verfaellie, 1988; Stone et al., 2001), and there are anecdotal reports that even a single exposure to a new face in the study phase of an experimental recognition memory paradigm can lead to differential SCR responses during subsequent recognition judgments (Morris et al., 2008).

Findings from experimental research with affective priming paradigms in healthy individuals suggest that autonomic arousal responses may even play a role in overt recognition judgments for the identity of faces (Goldinger and Hansen, 2005; Duke et al., 2014). For example, Goldinger and Hansen (2005) found that presenting a subtle vibrating tactile stimulus (a “buzz”) simultaneously with test items during a recognition memory test led to an increased endorsement of new faces as “old.” Following a similar rationale, Duke et al. (2014) demonstrated that the subliminal presentation of affective information (i.e., a happy face) prior to a test probe (i.e., an emotionally neutral face) increased the likelihood that participants judged the probe faces as familiar. These behavioral findings suggest that feelings of familiarity and corresponding overt recognition responses can be influenced by arousal. Such evidence is consistent with many other lines of evidence from the cognitive neuroscience literature at large, revealing that some internal bodily changes can be consciously experienced or “felt” (through interoceptive awareness), and influence conscious decision making (Critchley and Harrison, 2013). To the extent that abnormalities in autonomic arousal have been proposed to be a core feature of CS, the findings reviewed raise the possibility that overt judgments of person recognition may also be affected by these abnormalities in CS.

Most research and theoretical commentary on CS has focused on person recognition in the visual modality (i.e., face recognition). However, the generation of differential autonomic responses to famous individuals is not limited to faces but has also been shown to accompany voice recognition (Lewis et al., 2001). Lewis et al. (2001) reported larger SCRs for famous relative to non-famous voices, which were of comparable magnitude to the SCR response observed for famous versus non-famous faces in healthy individuals. In contrast to these findings with face- and voice cues, there is evidence to suggest that recognition of famous people based on their names is not accompanied by a differential autonomic response (Ellis et al., 1999). From this perspective, potential impairments in overt recognition of famous people in patients with CS, although perhaps not limited to faces, may still show some cue specificity.

Existing experimental evidence that speaks to the extent of overt person recognition impairments within and across modalities and cues in CS is currently limited. Several studies that have addressed this question by using faces of famous individuals have revealed some impairment (Young et al., 1993; Ellis et al., 1997; Breen et al., 2002; Lucchelli and Spinnler, 2008; Thiel et al., 2014). For example, Ellis et al. (1997) reported that two of four patients tested were impaired at judging whether a presented face was famous in a yes/no recognition task, and these same two patients also demonstrated deficits in identifying the occupation of the famous face in question. Particularly relevant to the current investigation, Lucchelli and Spinnler (2008) used a forced-choice recognition task to probe fame recognition in a patient with CS in association with an unspecified neurodegenerative condition; these authors observed a noticeable impairment in judging which of four faces was famous. It should be noted, however, that all of the aforementioned studies tested person recognition using faces only. Overt recognition of famous voices has only been examined in two individuals with CS in prior work (Reid et al., 1993; Lewis et al., 2001), with impairments observed in both cases. At present, it is unclear whether these impairments can be observed together, and whether they occur against a background of normal name recognition. Moreover, given that none of these prior studies compared performance of CS patients with that of patients of matched etiology, but no indication of CS symptomatology, it is also unclear whether the instances of impairment in overt person recognition that have previously been reported are in fact specific to CS.

A final question regarding the scope of person recognition impairments in CS is whether any such impairments extend beyond person identity and include deficits in overt recognition of facial affect, i.e., facial emotional expressions. Given the proposed role for abnormal autonomic responses in CS (Ellis et al., 1997; Hirstein and Ramachandran, 1997), it is conceivable that overt recognition of affect in others might also be impaired in CS patients. In fact, several researchers have suggested a potential link between the recognition of affect and the experience of affective states – a link that has been referred to as affective mimicry (e.g., Dimberg et al., 2000; Oberman et al., 2007; Heberlein et al., 2008; Stel and van Knippenberg, 2008). The limited research on recognition of emotional facial expressions in patients with CS has provided mixed results with respect to this issue. While some studies revealed no such deficit (Hirstein and Ramachandran, 1997), there are also reports of a modest deficit in identifying particular emotions such as fear or disgust (Breen et al., 2002). Prior studies typically required participants to discriminate between different types of emotions as opposed to detecting any sign of affect or judging the degree of emotional intensity. This is an important distinction to make, given that recent neuropsychological research in patients with focal lesions (but without any reported indication of CS) has shown that some prefrontal lesions can produce deficits that are only noticeable when fine-grained discrimination between subtle changes in facial expression within a given emotion category are required (Heberlein et al., 2008; Tsuchida and Fellows, 2012). At present, it is unknown whether CS may be associated with deficits in affect recognition of this nature.

### GOAL OF THE PRESENT STUDY

The goal of the current study was to shed more light on the nature and extent of overt impairments in person recognition uniquely associated with CS, focusing on the specific issues described above. We had a unique opportunity to pursue this goal by studying a patient with Dementia with Lewy bodies (DLB) who showed clear signs of CS and comparing him to another patient with DLB who did not experience CS, as well as to a group of healthy control participants. Although the majority of CS cases reported in the literature have been associated with psychiatric illness, affective disorders, focal lesions, or traumatic brain injury, recent epidemiological evidence suggests that CS and other related misidentification problems are also often seen as part of neurodegenerative disease, with a particularly large number of cases associated with DLB (for review, see Josephs, 2007; Harciarek and Kertesz, 2008; Devinsky, 2009; Thaipisuttikul et al., 2013). To address the extent of overt recognition impairment in the two DLB patients in our investigation, we administered fame-judgments for faces, voices, and names that involved assessment of familiarity as well as recovery of pertinent semantic knowledge. In addition, we sought to examine whether any deficits in person recognition would include problems in recognizing facial expressions of affect. To probe this ability, participants were asked to rate the intensity of fearful expression on a series of faces that varied in their intensity.

## MATERIALS AND METHODS

### PARTICIPANTS

#### DLB patients

**FIGURE 1:**
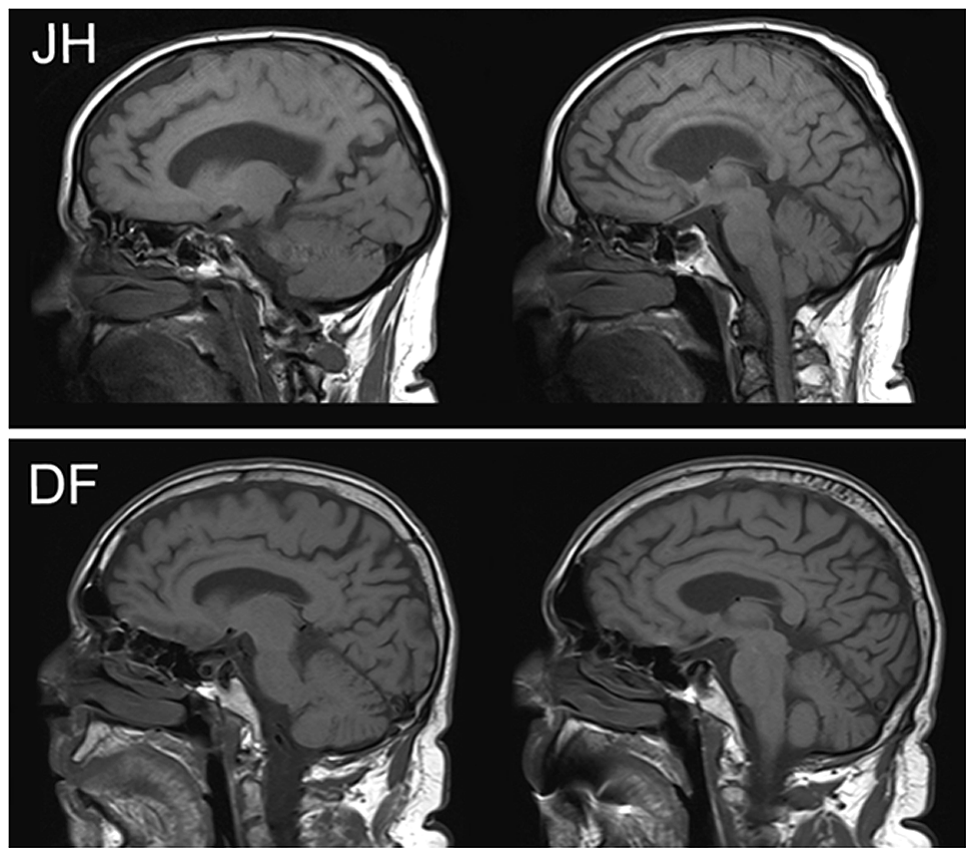
**Clinical MRI images in sagittal plane for patients JH (above) and DF (below).** There is visible atrophy in the dorsal aspects of mPFC for JH but not DF.

***JH (DLB w/Capgras).*** JH is a 79 year old male who met diagnostic criteria of DLB (McKeith et al., 2005; Ferman et al., 2011) at the time of testing. Specifically, this diagnosis was made on the basis of documented cognitive deficits in conjunction with the presence of well-formed visual hallucinations, spontaneous signs of Parkinsonism (masked facies), REM sleep behavior disorder, and neuroleptic sensitivity. Testing in the current study took place ∼1 year after the first report of cognitive difficulties. The patient had 9 years of formal education and additional vocational training. He primarily worked as a real estate sales agent prior to his retirement in 2003. The patient’s spouse of 28 years first noted signs of visual misperceptions about 1 year prior to testing. For example, JH reportedly saw a raging dog, and at another time a woman’s face, in the chandelier of their living room. Visual misperceptions also included hallucinations such as seeing a crack in the wall that required fixing. More recently he also reported signs of auditory hallucinations, such as a buzz that he thought was coming from an insect he could not see. First signs of CS were noticed by JH’s spouse ∼3 months prior to testing, and have reoccurred with considerable frequency across this period. These misidentifications always pertain to his spouse and follow the classic description of the Capgras delusion. Specifically, these incidents are characterized by the expressed belief that his spouse is not the person she claims to be but only looks similar to her (i.e., an imposter). When in an acute delusional state, JH is resistant to any change in his belief and is not receptive to rational counter-arguments or factual counter-evidence, such as the wedding band or a wedding photograph. When his son was present in one of the earliest instances of an acute Capgras delusion, JH even asked his son how he could be so sure that this was indeed his mother. The delusion is typically associated with some agitation and changed behavior, including active attempts to find his “true” spouse. It has led to many instances of marital conflict. Although the fully expressed delusion most frequently occurs in sleep-wake transitions and was not present at the time of testing, JH expressed in an interview that accompanied the testing session that his spouse never “feels the same” to him the way she used to. We take this phenomenological impression as a sign of a lasting cognitive deficit that is reflected in the experimental findings described here. JH has also reported misidentifications of place, specifically his home, at various times in combination with the Capgras delusion. Curiously, even when probed with specific cues provided by his spouse, he does not appear to recollect any episodes in which the Capgras delusion was acutely present. Visual inspection of a clinical magnetic resonance imaging (MRI) scan (see **Figure 1**) by a trained radiologist who was blind to the specific behavioral profile of this patient revealed mild atrophy of the medial frontal lobes and Sylvian fissures, with normal appearing posterior occipital and parietal regions. With respect to medication, JH was initially placed on a cholinesterase inhibitor (rivastigmine), and quetiapine (which was subsequently tapered off prior to testing) to help mitigate his delusions.

***DF (DLB w/o Capgras).*** DF is a 73 year old male who met diagnostic criteria of DLB (McKeith et al., 2005; Ferman et al., 2011) at the time of testing, which took place ∼1 month after his formal diagnosis of DLB, and 6 years after the onset of mild cognitive difficulties, as reported by his wife. He had 21 years of formal education, having completed a Bachelor’s degree as well as a Masters of Divinity and a Doctor of Divinity. He retired from his post as Pastor and community leader in 2008, after his spouse noted that he had been starting to forget things. Around this time, he also began to experience a variety of clinical symptoms including visuo-spatial difficulties, left hand tremor, rigidity, disturbed balance, REM sleep disturbances, and autonomic dysfunction, including orthostatic hypotension and urinary urgency/frequency. Based on a semi-structured interview conducted with his spouse, he reported experiencing hallucinations in 2012, in which he claimed to see and hear people and animals not currently present. Often he would act on these hallucinations, sometimes talking to the individuals he claimed to see. While he also experienced some paranoid beliefs, specifically sensing “someone following him,” these episodes were infrequent, and quickly resolved within a minute or so. His spouse also noted isolated incidents of visual misperceptions in DF that included, for example, mistaking their dog for a pair of shoes. Person recognition among family members was reported to be normal, although he sometimes apparently failed to recognize individuals he would have only been introduced to recently. His spouse also mentioned that he had experienced disorientation, and had reported difficulties with following directions. Furthermore, she noted changes in personality. In her view, he had turned from a very selfless and compassionate person into a somewhat selfish man who complains often. In social situations, he seemed to be unwilling to initiate or engage in any conversation unless it pertained to his immediate area of interest. Visual inspection of a clinical MRI scan (see **Figure 1**) by a trained radiologist revealed mild to moderate generalized atrophy, with slight predominance in temporal lobes. Similar to JH, DF was also prescribed a cholinesterase inhibitor (galantamine), and was additionally placed on dopamine (sinemet) and serotonin (citalopram) agonists to help alleviate Parkinsonism and depressive symptoms, respectively.

#### Neuropsychological profile

The results of several clinical neuropsychological tests of cognitive functioning for both patients are presented in **Table 1**. In terms of overall cognitive status as assessed using the Montreal Cognitive Assessment (MoCA; Nasreddine et al., 2005), both patients obtained scores in the range of mild cognitive impairment, with JH scoring slightly worse than DF. Both individuals showed clear signs of anterograde impairment in episodic memory, with the most pronounced deficits on non-verbal (visual) tasks. While JH performed numerically worse than DF on tests of recognition memory (Warrington word recognition; Warrington face recognition), DF obtained a lower score on the Benton face recognition test. Both patients were also impaired on perceptual tasks involving visual object processing, with particularly poor performance on the Embedded Figures task. On tests of executive functioning, specifically the Hayling and Brixton tests, both patients exhibited comparable levels of impairment.

**Table 1:** Neuropsychological profile of each patient.

	Patient JH	Patient DF
MoCA	21/30	24/30
Warrington face recognition (%ile)	<5th	10th
Warrington word recognition (%ile)	6–10th	50th
Doors and people (%ile)		
People test (immediate)	84th	75th
Doors test	25th	10th
Shapes test (immediate)	2nd	5th
Names test	50th	50th
Verbal memory	75th	75th
(people + names)		
Visual memory	5th	<5th
(doors + shapes)		
Benton face recognition	47 (normal)	37 (moderate impairment)
Hooper visual organization test	68 (normed score)	77 (normed score)
Embedded figures	1st quartile	1st quartile
Hayling sentence comprehension (scaled score)	3 (poor)	4 (low average)
Brixton spatial anticipation test (scaled score)	1 (impaired)	1 (impaired)

#### Healthy control participants

Ten control participants [all male; mean age=78.1 years (SD = 3.28); mean education = 11.7 years (SD = 2.83)] were recruited to participate in various aspects of the current study. They were selected to match the patient with CS symptomatology (i.e., JH) in sex, age, and years of education as closely as possible. Of these 10 participants, nine individuals completed the famous faces (Experiment 1A) and famous names (Experiment 1C) tasks, with eight participants completing the famous voices (Experiment 1B) task as well. All ten participants completed the fear rating task (Experiment 2). Controls were screened to ensure the absence of current or past neurological or significant psychiatric disorders. This research project was conducted with the approval of the Health Sciences Research Ethics Board (HSREB) at Western University, and all participants gave written informed consent.

### EXPERIMENTAL PROCEDURES

#### Experiment 1A: famous face recognition

We created a list of 64 famous faces and 64 faces of non-famous individuals. Famous individuals were sampled broadly so as to increase the likelihood that they would indeed be known to study participants. Each famous person belonged to one of three discrete historical eras (1950 to 1969, 1970 to 1989, and 1990 to 2009) and to one of four occupation categories (politicians, movies actors, television actors/personalities, and athletes), with roughly equal numbers of famous faces in each era and category. Images of famous faces from a front view were retrieved through a Google Image search and the Life magazine image archive^1^. Each famous face was yoked with a non-famous face that was found via a Google Image search. Non-famous faces were matched to the famous individuals’ sex, approximate apparent age, and era. In order to ensure a lack of fame for the non-famous faces, images of anonymous models from advertisements and images from out-of-country real estate brokerages, barristers, and genealogical websites were used. If a name was displayed by the search engine, we also checked that it did not point to a famous person. All color images were transferred to grayscale and all images were standardized in size to be 380 pixels in height and between 204 and 302 pixels in width. For each image, we superimposed a white oval frame around the face to occlude scene background and clothing.

**FIGURE 2:**
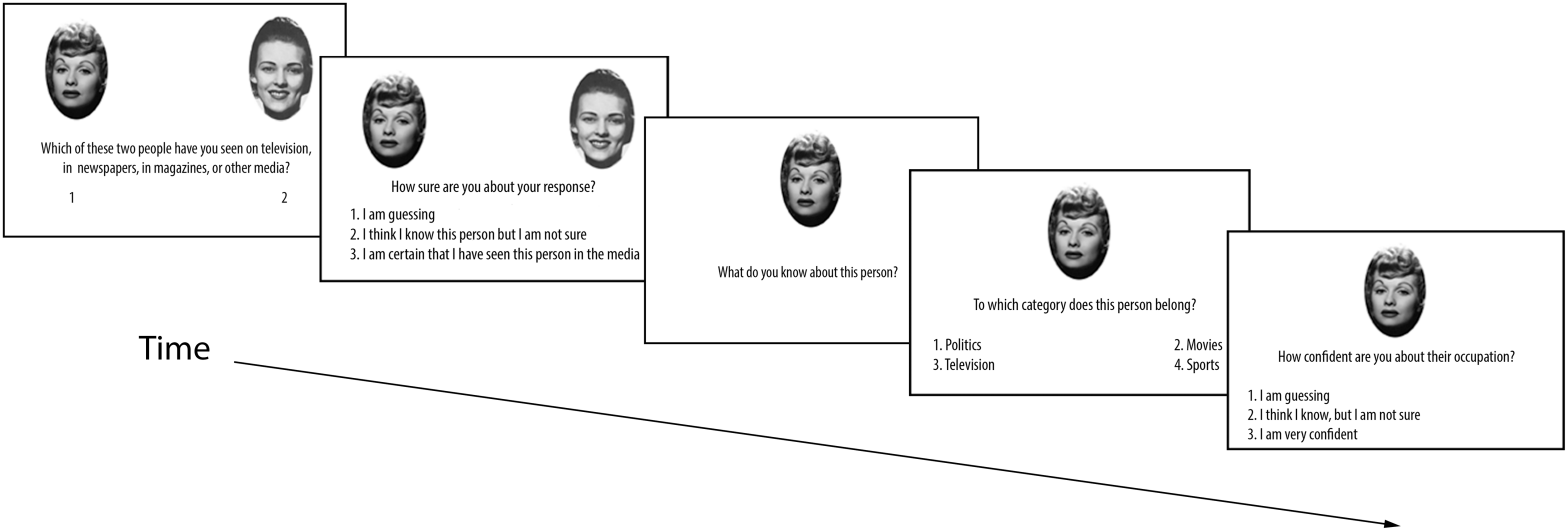
**Sequence of events for each trial in Experiment 1A.** Experiments 1B and 1C use a similar trial structure (see text).

The experiment was presented on a laptop computer using E-prime 1.1 programming software. Participants provided the experimenter with oral responses to each screen prompt, and the experimenter entered numeric responses by keyboard press and recorded any knowledge generated by participants. Trials were presented in random order. For each trial, participants viewed one famous face and its non-famous yoke on the screen, side by side (see **Figure 2**). For 50% of all trials, the famous face was on the left side. While the image pairs were on screen, participants verbally indicated which of the two faces they knew from the media. Both faces remained on the screen as participants were asked to rate their confidence in their choice on a scale of one to three, corresponding to “I am guessing,” “I think I know, but I am not sure,” and “I am certain that I have seen this person in the media,” respectively. Following this response, the face selected as famous remained on the screen, and the participant was asked to provide information (if any) they could recall about that person, which the experimenter recorded. Participants then completed a forced-choice occupation judgment for the chosen individual (with the face still on the screen) from the four different categories, and rated their confidence for this decision. There were no time restrictions for any portion of these trials.

#### Experiment 1B: famous voice recognition

This task was administered in the same testing session as the experiment on face recognition. Therefore, we created a new list of 48 famous people to avoid overlap. Identities were selected based on criteria similar to those of Experiment 1A. Voices of famous and non-famous people were extracted from interviews available online (at YouTube.com) and were selected with the goal of minimizing any sound context. In addition, we aimed to minimize recognition of voice based on speech content (e.g., an actor talking about his movie). Non-famous voices were extracted from news interviews, television game shows, and documentary interviews. Where possible, the non-famous person’s name was verified as not being associated with any fame. Prior to testing, pilot work was conducted based on transcripts of all voice clips to ensure that participants could not identify the famous person based on content. Voice clips were edited in Audacity 1.3. Extraneous noise, distortion, and non-verbal vocalizations were removed or reduced. Due to the age of some voice clips, we also matched non-famous clips on sound quality to assure that the voice pairs had equal levels of distortion that might hint at a particular era. Pairs of voice clips were matched in length within 1 s. Voice clips varied in duration between 7 and 12 s, with a mean duration of 8.71 s (SD = 1.29).

The trial structure for the famous voices task was comparable to that for the famous faces task in Experiment 1A. Members of pairs of famous and non-famous voices were presented sequentially and order varied across trials. Participants listened to the voice clips through the speakers on a laptop. Participants were alerted to any upcoming trial with the prompt, “Ready?” For 50% of the trials, the first voice was that of the famous person. Participants were prompted to decide which of the two voices belonged to a famous person. Participants were offered an option to replay the two voice clips as often as they wished. Following the famous voice decision, participants were asked to rate their confidence in their decision. Following semantic knowledge generation, participants heard the selected voice clip once more and were then prompted to make a forced-choice occupation decision followed by a confidence judgment for that decision. At the conclusion of each trial, participants had the option to take a momentary break prior to the next trial.

#### Experiment 1C: famous name recognition

The names of the famous individuals from the famous faces task were used as famous names in the current task. This approach allowed for a direct comparison of recognition performance across modalities while maintaining equivalent demands in semantic knowledge. To minimize any carry-over effects, testing of names and faces was separated by at least a 1 week delay in all study participants. Non-famous first and last name combinations were generated to match each famous name for syllable count and name frequency based on the 1990 US Census Database^2^. First names were matched for gender. If either the first or last name of a famous person did not occur in the 1990 US census database, then genealogical web sites pertaining to the name’s origin were consulted and a non-famous name was generated such that it matched the syllable count of its famous counterpart (e.g., “Marenka Koupilova” was a non-famous corresponding item to “Martina Navratilova”). We confirmed that there was no level of fame associated with these names by performing Google and Wikipedia searches.

The trial structure in this experiment was identical to that of the Famous Faces task, except that each face stimulus was replaced by a name presented as text.

#### Experiment 2: fear expression rating

Stimuli consisted of colored images of faces taken from the Karolinska Directed Emotional Faces database (KDEF) as well as the NimStim Emotional Face Stimuli database (Lundqvist et al., 1998; Tottenham et al., 2009). All faces were cropped down to a specific oval template, including the forehead, eyes, nose, mouth, and full jaw, while leaving out hair, jewellery, and ears. All face stimuli were surrounded by a rectangular background of Gaussian noise. The faces were binned according to the intensity of the fearful expressions into low (14 faces), moderate (15 faces), and high (16 faces) groups, as established by previous ratings from an independent sample of participants. During each trial, participants fixated on a central cross for a period of 2000 ms, followed by a 1500 ms presentation of the face. After 1500 ms, a rating scale appeared below the face (which remained on the screen). Participants were asked to judge how fearful the depicted expression was on a 6-point scale, with 0 representing “no fear” and 6 representing “very fearful.” Judgments were made in a self-paced manner.

## RESULTS

To compare recognition performance of each patient to that of healthy age-matched control participants, we used Crawford’s modified *t*-test (Crawford and Howell, 1998) for all comparisons. This test statistic is often preferred over the standard *t*-statistic when the performance of a single patient is being compared to that of a small control sample, as it corrects for potential bias in parameter estimates of the population standard deviation that is inherent to small samples. It should be noted that this statistical test is generally considered to be conservative in establishing abnormalities.

### EXPERIMENT 1A: FAMOUS FACE RECOGNITION

**FIGURE 3:**
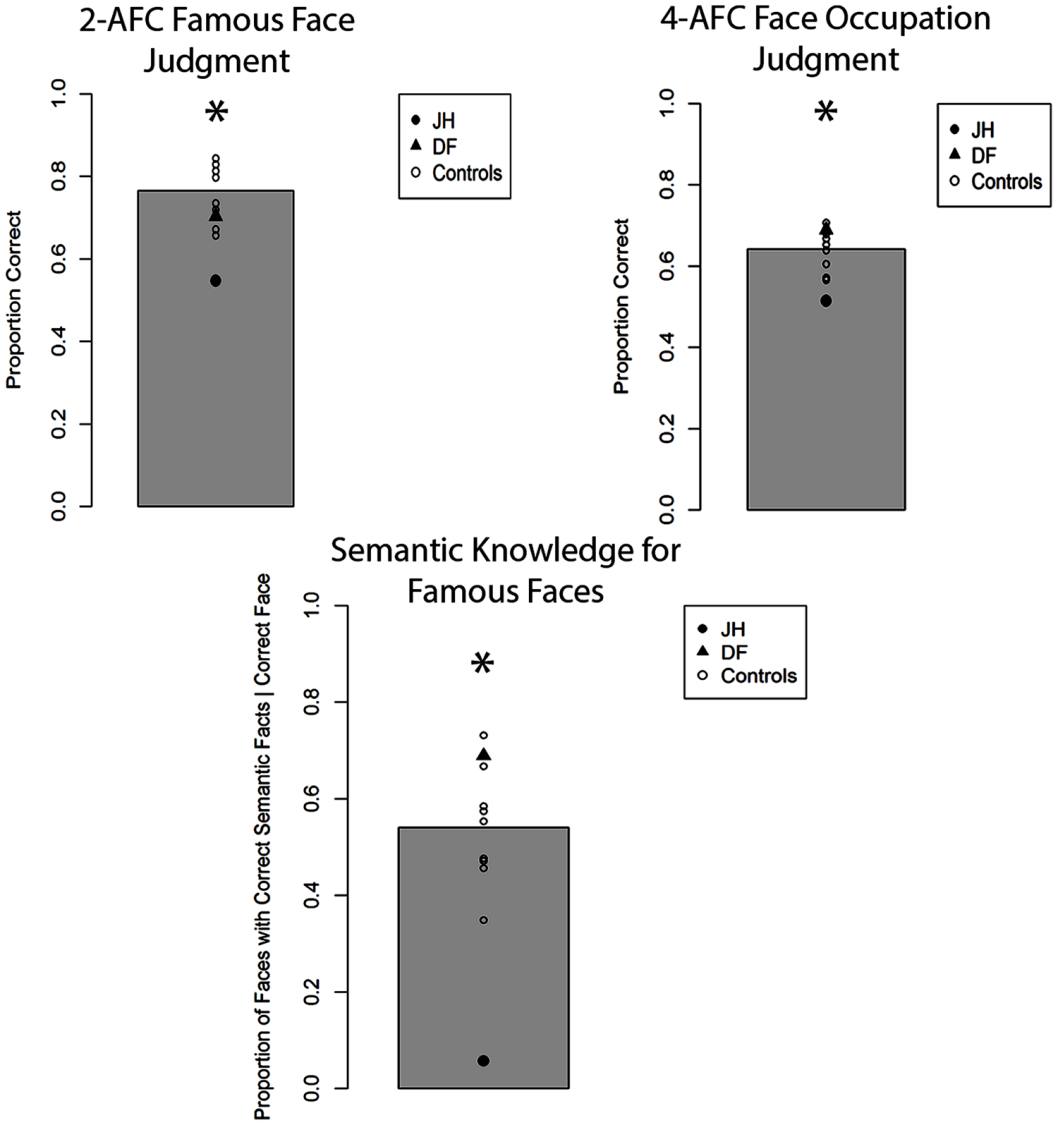
**Accuracy for two alternative forced-choice (2-AFC) fame recognition judgments, corresponding occupation judgments, and semantic knowledge generation for famous faces in Experiment 1A.** Gray bar represents the mean of controls. *p < 0.05 for JH relative to controls.

The mean proportion of correct responses for famous face recognition judgments for all participants is depicted in **Figure 3**. Comparing this proportion for patient JH and controls revealed a marked impairment, *t*(8) = 2.90, *p* = 0.01 (one-tailed), *d* = 3.06. In contrast, patient DF’s performance was comparable to that of controls, *t*(8) = 0.83, *p* = 0.43, *d* = 0.87, with no observable impairment. To evaluate participants’ confidence for recognition judgments, we calculated the proportion of “certain” responses made for all recognition decisions. We found that JH tended to give fewer “certain” responses relative to controls, *t*(8) = 1.74, *p* = 0.06 (one-tailed), *d* = 1.83, with DF performing in the normal range, *t*(8) = 1.20, *p* = 0.27, *d* = 1.26. In fact, JH responded with “certain” only twice across all trials, whereas the mean number in controls was 18 (out of 64 trials). To examine the extent to which participants were able to provide accurate semantic information about correctly chosen famous faces, we calculated the proportion of such famous faces for which any correct semantic information could be generated. Again, we found that JH’s performance was impaired, as he produced semantic information to fewer of the correctly chosen famous faces, *t*(8) = 3.94, *p* = 0.002 (one-tailed), *d* = 4.16. Again, DF did not differ from control participants on this measure, *t*(8) = 1.21, *p* = 0.26, *d* = 1.28. The same pattern of performance across participants emerged when we examined the proportion of accurate forced-choice occupation judgments for correctly chosen famous faces, with JH, *t*(8) = 2.37, *p* = 0.023 (one-tailed), *d* = 2.50, but not DF, *t*(8) = 0.87, *p* = 0.80, *d* = 0.92, being impaired relative to controls. Finally, JH *t*(8) = 1.73, *p* = 0.06 (one-tailed), *d* = 1.83, but not DF *t*(8) = 0.25, *p* = 0.81, *d* = 0.27, showed reduced confidence in these occupation judgments, as reflected in the proportion of “certain” response given across all trials.

### EXPERIMENT 1B: FAMOUS VOICE RECOGNITION

**FIGURE 4:**
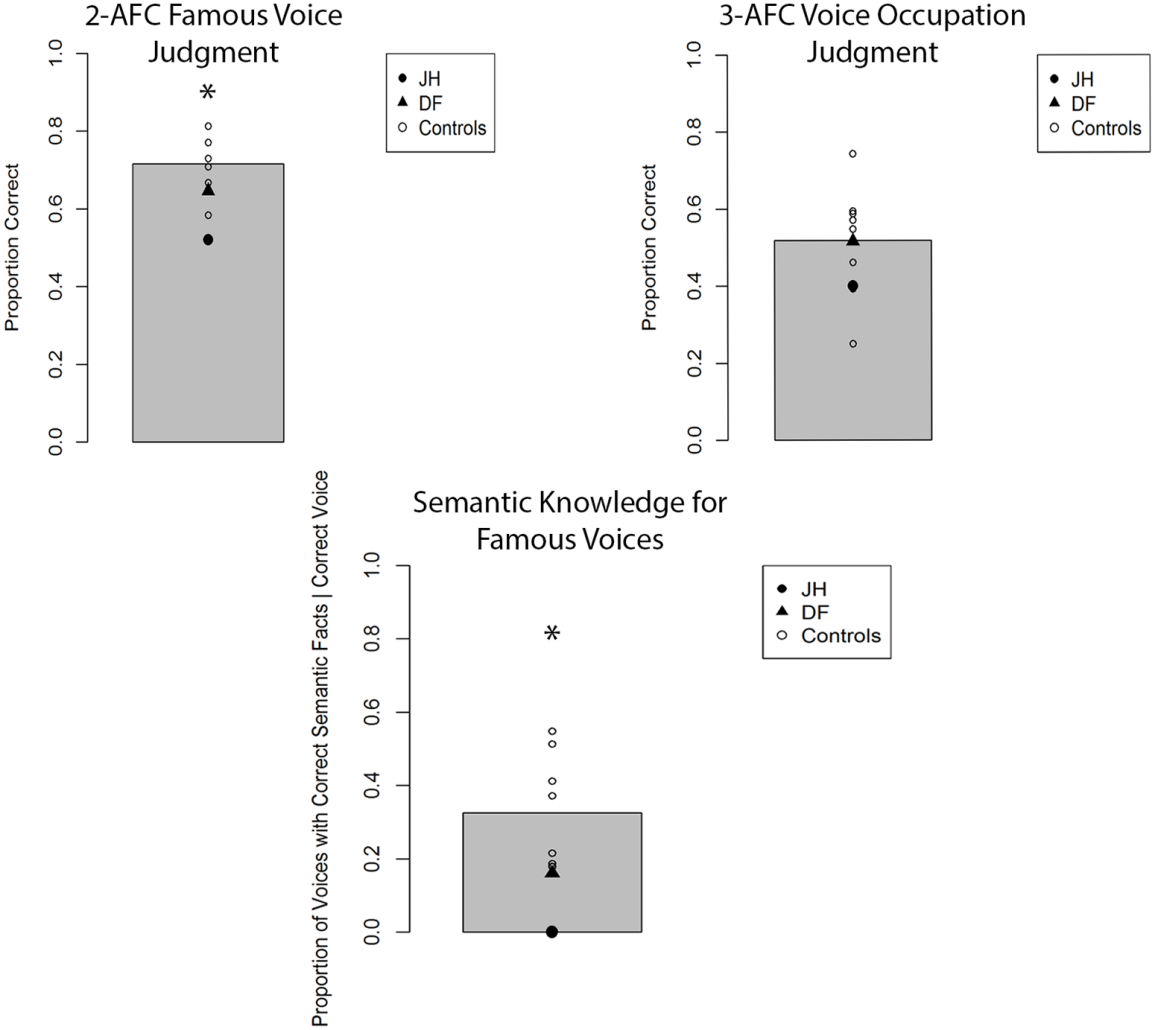
**Accuracy for two alternative forced-choice (2-AFC) fame recognition judgments, corresponding occupation judgments, and semantic knowledge generation for famous voices in Experiment 1B.** Gray bar represents the mean of controls. *p < 0.05 for JH relative to controls.

The mean proportion of correct responses for famous-face recognition judgments for all participants is depicted in **Figure 4**. In comparing the proportion of correct fame decisions based on voice cues for patients and controls, we found that JH (0.52) exhibited an impairment, *t*(7) = 2.25, *p* = 0.02 (one-tailed), *d* = 2.39, while DF (0.65) performed in the normal range (*M* = 0.71), *t*(7) = 0.81, *p* = 0.44, *d* = 0.86. Given that the overall level of performance and confidence for fame judgments was lower for all participants with voice cues, we collapsed “certain” and “not sure” responses to obtain an index of high confidence for fame judgments. Again, JH (0.00) tended to exhibit less confidence than healthy controls (*M =* 0.32), *t*(7) = 1.58, *p* = 0.07 (one-tailed), *d* = 1.67, while DF’s confidence (0.06) did not differ, *t*(7) = 1.24, *p* = 0.24, *d* = 1.35. In terms of semantic knowledge, the proportion of correctly selected famous voices for which JH (0.00) could recall any correct semantic information was smaller than that of controls as well (*M* = 0.33), *t*(7) = 1.98, *p* = 0.04 (one-tailed), *d* = 2.10. By contrast, DF was able to retrieve semantic information for a similar proportion (0.16) of correctly selected voices as controls, *t*(7) = 1.00, *p* = 0.35, *d* = 1.06. When we examined the proportion of accurate forced-choice occupation judgments for correctly selected famous voices, neither JH, *t*(7) = 0.75, *p* = 0.24 (one-tailed), *d* = 0.80, nor DF, *t*(7) = 0.02, *p* = 0.99, *d* = 0.02, differed from controls. However, this result must be interpreted with caution given the overall low level of performance (see **Figure 3**). As with faces, we found that JH’s, *t*(7) = 2.07, *p* = 0.039 (one-tailed), *d* = 2.19, but not DF’s, *t*(7) = 1.55, *p* = 0.16, *d* = 1.65, confidence in occupation judgments was reduced.

### EXPERIMENT 1C: FAMOUS NAME RECOGNITION

**FIGURE 5:**
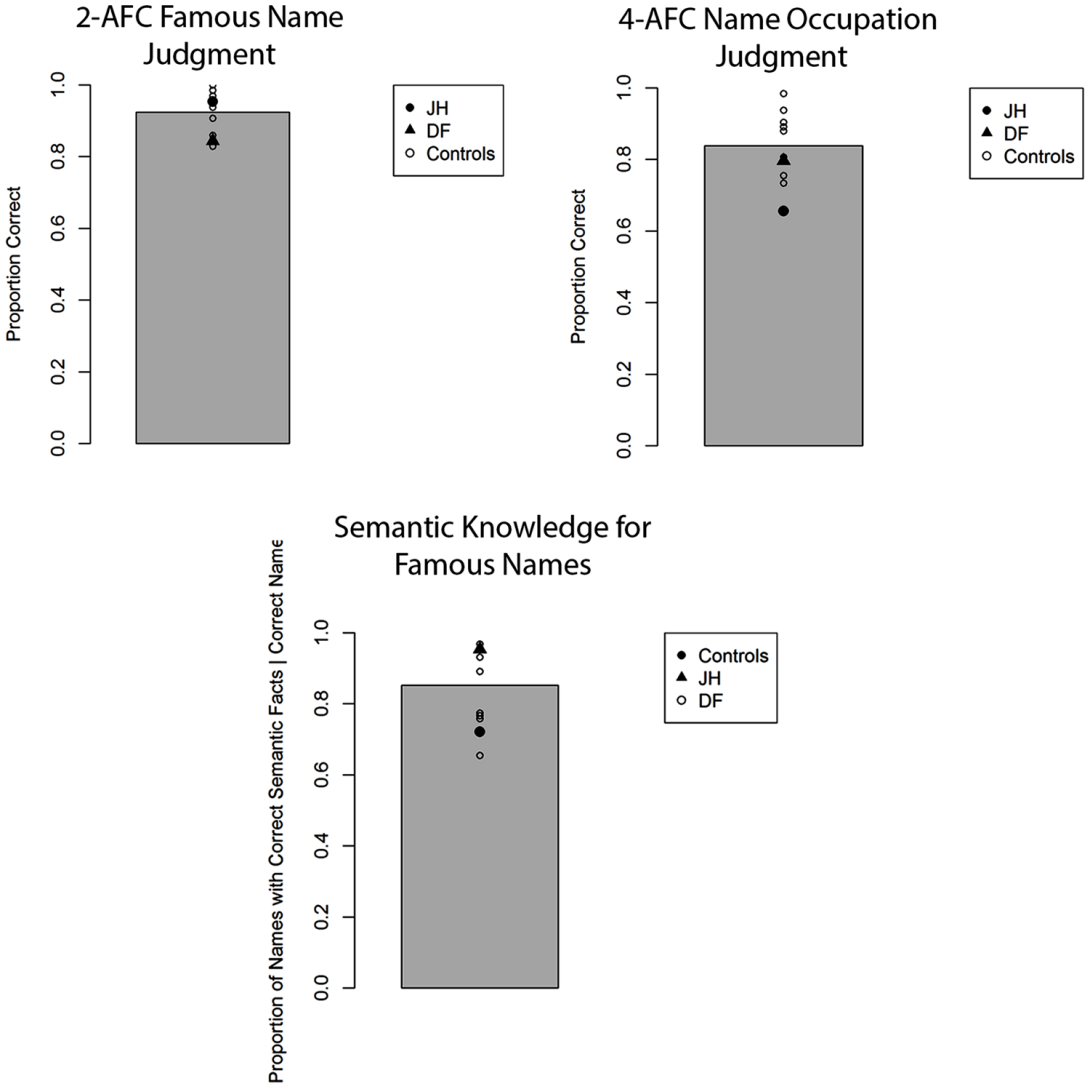
**Accuracy for two alternative forced-choice (2-AFC) fame recognition judgments, corresponding occupation judgments, and semantic knowledge generation for famous names in Experiment 1C.** Gray bar represents the mean of controls.

The mean proportion of correct responses for famous-name recognition judgments is depicted in **Figure 5**. Notably, JH correctly recognized a similar proportion of famous names as control participants on this task, *t*(8) = 0.45, *p* = 0.66, *d* = 0.47, with DF also showing no signs of impairments in recognition accuracy, *t*(8) = 1.21, *p* = 0.26, *d* = 1.27. Confidence for correctly selected names was evaluated by comparing the proportion of “certain” responses made for each trial. JH demonstrated similar levels of confidence as controls, *t*(8) = 0.32, *p* = 0.75, *d* = 0.34, as did DF, *t*(8) = 0.63, *p* = 0.54, *d* = 0.67. The proportion of famous names for which any correct semantic information was generated was similar for JH and controls, *t*(8) = 1.07, *p* = 0.31, *d* = 1.13, as well as for DF and controls, *t*(8) = 0.84, *p* = 0.43, *d* = 0.89. Similarly, we found no evidence of impairment for the forced-choice occupation judgments for correctly selected famous names provided by either JH,* t*(8) = 1.61, *p* = 0.15, *d* = 1.70, or DF, *t*(8) = 0.37, *p* = 0.72, *d* = 0.39. Finally, confidence in these occupation judgments did not differ between JH and controls, *t*(8) = 0.36, *p* = 0.73, *d* = 0.38, nor DF and controls, *t*(8) = 0.51, *p* = 0.62, *d* = 0.54. Together, there was no indication of abnormalities in person recognition based on names in either patient. An important aspect of these results, then, is that JH could recognize the names of the same individuals whose face he could not recognize accurately in Experiment 1A. As an aside, we also note that this preservation of semantically based name-recognition contrasts with JH’s deficits on neuropsychological tasks of episodic memory for words. This latter dissociation is in line with many prior findings demonstrating independence between deficits in episodic versus semantic memory (e.g., Vargha-Khadem et al., 1997; Hodges and Graham, 2001; Tulving, 2002).

### EXPERIMENT 2: FEAR EXPRESSION RATING

**FIGURE 6:**
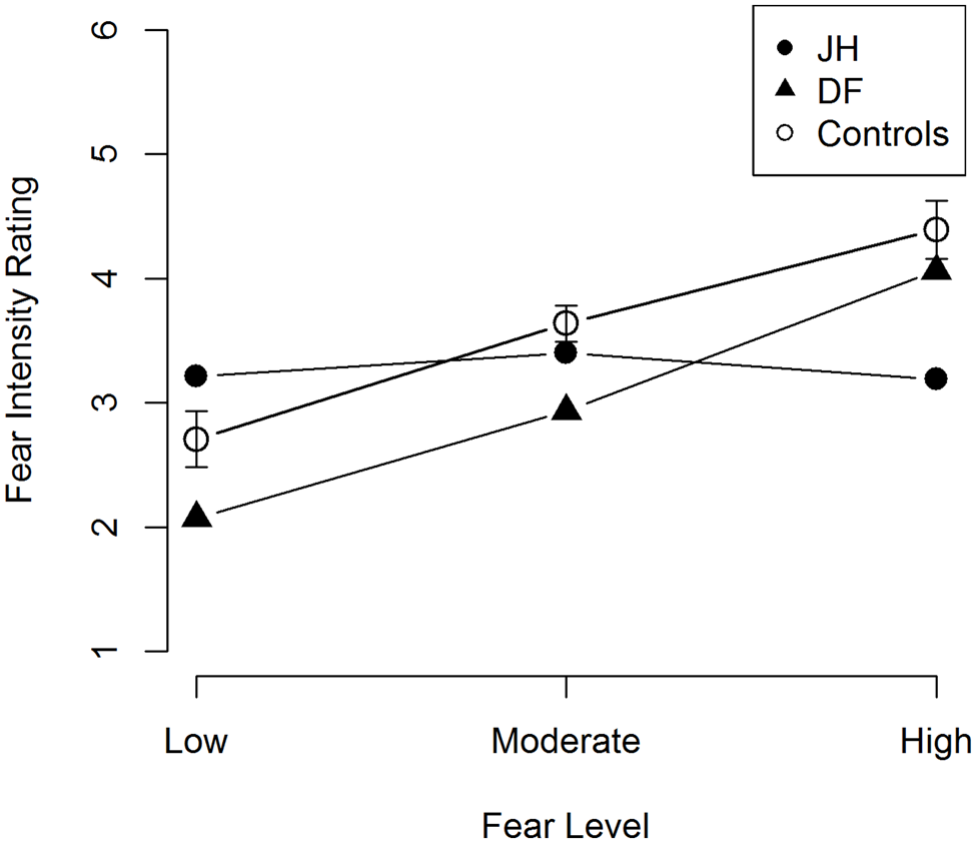
**Mean fear intensity ratings for fearful faces.** Error bars represent the standard error of the mean for control participants.

Our primary interest for this task was to determine whether JH’s perceived intensity of emotional expression differed from that of controls in terms of its coupling with manipulated (i.e., depicted) intensity. To this end, we performed a linear trend analysis in each patient and in healthy control participants. Healthy controls exhibited a significant linear trend, *t*(9) = 4.02, *p* = 0.002, *d =* 1.29, with fear ratings being positively related to the intensity of the fearful expression (see **Figure 6**). Patient DF also exhibited a similar linear trend, *F*(1,42) = 21.0, *p* < 0.001, *R^2^_contrast_* = 0.33. By contrast, patient JH did not exhibit a significant linear trend, *F*(1,42) = 1.39, *p* = 0.25, *R^2^_contrast_* = 0.032, suggesting that his perceived fear intensity was decoupled from the depicted intensity level.

## GENERAL DISCUSSION

The current case study aimed to examine the scope of overt person recognition impairments associated with CS in DLB, focusing on both the recognition of identity and affect as conveyed through facial expressions. Experiment 1 probed recognition of person identity across three types of cues (faces, voices, and names, respectively) with two alternative forced-choice (2-AFC) fame judgments. Relative to healthy age-matched controls, the DLB patient with CS (JH), but not the patient without CS (DF), exhibited an impairment in recognizing famous faces and voices. JH also showed reduced confidence in these judgments and was found to be impaired at accessing pertinent semantic knowledge in response to face and voice cues; specifically, he generated fewer semantic facts, and was less accurate in judging the occupation of correctly chosen famous faces and voices. This pattern of results stands in contrast to the pattern we observed when person recognition was probed with names. Here, JH performed as well as DF and healthy controls and showed normal levels of confidence in his fame judgments. There was also no evidence of any semantic impairment with name cues. Together, these results reveal noticeable cue-specific deficits in overt recognition of person identity that are associated with CS. In Experiment 2, we also found evidence for impairments in overt judgments of facial expressions. Unlike DF and healthy controls, JH provided fear ratings that did not vary with the depicted intensity of fearful expression.

According to what is perhaps the most prominent account of the Capgras delusion in the current literature, perceptual representations of familiar faces are decoupled from the appropriate affective signals that imbue faces with a “glow” as a marker of familiarity (Hirstein and Ramachandran, 1997; Breen et al., 2000; Ellis and Lewis, 2001; Young, 2008). Given extant evidence that points to a role for autonomic signals in person recognition beyond spouses and close relatives (Stone et al., 2001; Vico et al., 2010; Guerra et al., 2012), and given the previously reported influences of arousal on overt recognition memory judgments (Goldinger and Hansen, 2005; Morris et al., 2008; Duke et al., 2014) we predicted that CS would be associated with subtle impairments in overt recognition of people other than those targeted by the delusion. Indeed, we found evidence for such impairments in fame judgments based on faces and voices, with a preservation of name recognition. Inasmuch as prior research suggested that autonomic responses (as measured with SCR) are associated with recognizing faces and voices (Ellis et al., 1997; Hirstein and Ramachandran, 1997; Lewis et al., 2001) but not names (Ellis et al., 1999), the behavioral dissociation we observed is consistent with the presence of abnormal autonomic arousal signals in JH. As such, it is also consistent with past research findings pointing to autonomic dysfunction in DLB (e.g., Thaisetthawatkul et al., 2004). We acknowledge, however, that we can only claim consistency given that we did not include psychophysiological measurements of autonomic responses in our experiments. Nevertheless, the fact that we observed this behavioral dissociation even though recognition of the same famous individuals was probed with face- and name cues, suggests that it is clearly not an amodal representation of person knowledge that is impaired in patient JH.

As noted in the Introduction, prior research that systematically addressed overt person recognition in CS is scarce. The handful of studies relevant to this issue have, in their majority, documented hints of impairment when famous faces were used as cues (Young et al., 1993; Ellis et al., 1997; Lewis et al., 2001; Lucchelli and Spinnler, 2008; Thiel et al., 2014, but see Breen et al., 2002). The tasks employed to probe person recognition and the etiology that caused CS, however, differed considerably across studies. Given the etiology of JH’s neurological condition, the findings reported by Lucchelli and Spinnler (2008) appear most relevant. These authors examined a patient in whom CS was associated with a neurodegenerative condition (of unknown origin). Like patient JH in the current study, the individual exhibited marked impairments in making forced-choice fame judgments for faces. Curiously, when the name of the famous individual was presented as an additional cue, the patient was able to use this information to select the famous face among the different alternatives. Thus, in line with the current findings, these prior results also point to some preservation of name-recognition abilities in CS. This preservation may hold utility in developing rehabilitative strategies aimed at mitigating the person recognition difficulties expressed in other modalities (i.e., faces, voices; see Cousins, 2013, for potential directions).

The majority of past studies that probed person recognition in CS have relied on tests involving faces as cues. To our knowledge, only one study addressed both face and voice recognition in the same patient (Lewis et al., 2001). These authors reported a case with voice-specific CS who was impaired in recognizing famous voices only, and whose autonomic abnormalities captured with psychophysiological recordings were also limited to voices. Such findings could suggest that the proposed disconnection between perceptual person representations and affective responses in CS does not always affect multiple modalities. The specific nature and extent of the structural lesion that is present in any given case of CS will likely determine whether the functional deficit affects person recognition based on one or more modalities and types of cues. In DLB, the modality in which hallucinations and/or misperceptions occur may be predictive as to whether person recognition impairments will be uni- or multi-modal. While visual hallucinations appear to be present in all cases of CS associated with DLB, reported auditory hallucinations are more variable (Ferman and Boeve, 2007; Josephs, 2007; Thaipisuttikul et al., 2013). Against this background, it is informative that JH experienced hallucinations in both modalities at the time of testing.

Given the central importance of the distinction between overt and covert responses in the literature on CS and prosopagnosia, an important issue to consider is whether the tasks we used to probe recognition of famous faces and voices in the current study do indeed reveal impairments in overt, i.e., conscious, person recognition. In experimental research on recognition memory conducted with study-test paradigms, it has been argued convincingly that performance on forced-choice tasks does not always rely on conscious access to stored information, and that implicit memory processes can sometimes drive behavioral responses (Voss et al., 2008; Paller et al., 2009; Dew and Cabeza, 2011; see also Bowles and Köhler, 2014 for discussion in relation to fame judgments). By this view, the recognition impairments we observed in association with CS in the present study could be seen as a covert expression of the presumed deficit in autonomic responding. There is other evidence in the current set of findings, however, that speaks against such an account. Critically, JH’s forced-choice recognition accuracy for fame judgments was related to his expressed confidence in the judgments. Specifically, the recognition deficits we observed for faces and voices, unlike the preserved recognition judgments for famous names, were associated with reduced levels of subjective confidence in the corresponding fame decisions when compared to healthy participants. Such a correspondence between subjective confidence and objective accuracy is typically interpreted as evidence that argues for a role of conscious awareness in the decisions at hand (see Dienes, 2008; Bowles and Köhler, 2014, for further rationale). As such, it suggests that the impairment we observed does indeed extend to overt person recognition. That we also observed noticeable deficits in the generation of semantic knowledge in response to face and voice cues, but again not names, provides further strong support for this interpretation.

In the present study, we also found impairments in the perception of affective information as conveyed through facial expressions in patient JH. Prior research on this issue in other CS cases has provided somewhat equivocal findings, with a handful of studies reporting no deficit (Hirstein and Ramachandran, 1997; Lucchelli and Spinnler, 2008; Thiel et al., 2014) and others revealing modest deficits in (Young et al., 1993; Breen et al., 2002). While differing etiologies could at least in part account for these mixed results, and for discrepancies with the current findings, differences in the type of affective judgments employed warrant particular consideration. The method used in prior studies that revealed no impairment in association with CS (Young et al., 1993; Hirstein and Ramachandran, 1997; Breen et al., 2002; Thiel et al., 2014) required participants to identify the category of emotional expression depicted (e.g., fear versus happiness). By contrast, in the current study, we revealed deficits by asking participants to rate the intensity of the emotion displayed within a given category, specifically fear. Recent neuropsychological evidence suggests that neural mechanisms in prefrontal cortex that support fine-grained intensity judgments can be dissociated from those that allow for discrimination between different categories. In particular, the former but not the latter type of affective judgment has been shown to be impaired following damage to vmPFC (Heberlein et al., 2008; Tsuchida and Fellows, 2012), a cortical region that has also been implicated in the control of autonomic responses in prior research (Tranel and Damasio, 1994; Bechara et al., 1999; Critchley et al., 2003; Williams et al., 2005; Medford and Critchley, 2010). We note that clinical visual inspection of MRI scans revealed atrophy in medial prefrontal cortex (mPFC) in JH. Thus, it is possible that the deficit in overt recognition of affect intensity we observed in JH is related to the abnormal autonomic responses in person recognition that have informed much research on CS over the past two decades. Evidence to support such a link comes from research in healthy individuals showing that affect intensity judgments for faces are sensitive to interoceptive cues that arise from afferent cardiovascular feedback (Gray et al., 2007, 2012). Future research with psychophysiological recordings could address the proposed relationship between deficits in these domains in CS more directly.

Perhaps the most puzzling aspect of CS is that patients’ delusions are restricted to one individual who always shares close emotional bonds with the patient, most frequently the spouse. Given the recognition impairments for famous faces and voices that were associated with CS in the present study, one might wonder why the patients’ delusions do not include other personally familiar or famous individuals. In order to answer this question, it is helpful to consider models that attribute delusions to noisy prediction error signals that engender false inferences (Kapur, 2003; Fletcher and Frith, 2009; Corlett et al., 2010 but see Griffiths et al., 2014 for current limitations). These models adopt a Bayesian framework that emphasizes the role of expectation in guiding perception and inference, and suggest that delusions may arise when current experience is deemed inconsistent with prior expectations. In the case of CS, autonomic dysfunction has been proposed to be associated with a phenomenological experience that differs from the one the patients would have typically had for many years in interactions with their spouse or loved ones, and that would therefore produce a salient prediction error; adopting the belief that the spouse is an imposter could resolve this mismatch between expectation and experience (Young, 2008; Corlett et al., 2010). In this account, the specificity of the patients’ delusions may arise because encountering a loved one is associated with a much stronger expectation of an accompanying autonomic signal than encountering other familiar individuals. Indeed, recent psychophysiological evidence has shown that affective responses to loved ones are associated with greater arousal and positive valence than those to other familiar individuals (i.e., famous people), as measured by SCRs and zygomatic muscle activity, respectively (Vico et al., 2010; Guerra et al., 2011). Moreover, these psychophysiological measures have also been shown to be greater for romantic partners as compared to family members (e.g., parents).

A critical aspect of the design of the current study was that we compared a patient with CS to another individual who did not report CS, but suffered from the same type of neurological condition, i.e., DLB. We included this comparison in an effort to reveal impairments that are uniquely associated with CS, while aiming to control for other cognitive and behavioral effects that are associated with DLB more broadly (see Collerton et al., 2003; Metzler-Baddeley, 2007; Johns et al., 2009, for review). This type of comparison seems particularly important when testing patients with neurodegenerative conditions, which are known to affect cognitive functioning more broadly than focal lesions (see Jedidi et al., 2013 for similar design in a single-case study on CS in Alzheimer’s disease). Although the results from the present single-case study clearly advance our understanding of the nature of person recognition impairments associated with CS, we recognize that further research will be needed to examine the extent to which they generalize to other CS patients with DLB and other etiologies.

We note that although the two DLB patients tested here were well matched in terms of general level of cognitive functioning, as reflected in their MOCA scores, there were noticeable differences in formal education. It seems unlikely, however, that these differences in educational background could account for the differences in person recognition we observed between these two individuals, given that JH was able to accurately recognize the names of the same famous individuals for whom his face recognition was poor. This result suggests that JH does indeed possess intact semantic representations of the individuals in question, but that he was unable to access these representations when presented with faces. In addition, two of the healthy age-matched control participants tested here shared the same level of formal education as JH. Given that one of them was the most accurate of the control participants in recognizing famous faces, with the other performing similar to DF in the middle range, it seems unlikely that level of education is a critical determinant of high performance in this task. It is also not clear how reference to educational background could explain why impairments in recognition of affect intensity were present only in JH and not DF, as this task employs subjective ratings and does not depend heavily upon knowledge acquired through formal education. In a similar vein, we also note that the two patients did not differ in their neuropsychological test scores of face recognition and visual object processing. Accordingly, we argue that the differences between both patients, in terms of the presence of CS and associated cognitive deficits, reflect variability that is the result of differential vulnerability of different brain regions to DLB pathology across individuals, a conclusion that is also supported by the clinical MRI data presented here.

Recent findings from a large-scale neuroimaging study on the neural correlates of psychotic symptoms in DLB speak to the link between atrophy in different cortical regions and variability in behavioral profile (Nagahama et al., 2010). One of the regions that showed hypo-perfusion in relation to the presence of misidentification syndromes in the sample of patients examined was the insular cortex. This finding is of particular relevance to the current investigation given that the anterior insula, together with mPFC and regions of the anterior cingulate cortex, have been implicated in the control of autonomic functions and in interoceptive awareness (see Critchley and Harrison, 2013, for recent review), and may be related to the pattern of mPFC atrophy observed in patient JH. On another level, this atrophy and the reported presence of a consistent patterns of hypo-perfusion across multiple DLB patients with misidentification syndromes (Nagahama et al., 2010) lends support to the notion that, although the critical delusion that defines CS may not be continuously present, there are lasting neuropathological changes that underlie the cognitive abnormalities that are unique to CS, and that may be necessary for the acute expression of this delusion.

Taken together, the findings reported in the present investigation demonstrate that deficits in person recognition associated with CS extend to persons that are not targeted by the delusion. Interestingly, these impairments were observable in overt recognition judgments, and pertained not only to recognition of identity but also to recognition of affect intensity. While we have emphasized that this overall pattern of results can be accommodated, and even be predicted, based on theories that place deficits in autonomic responding to perceptual person cues at the core of CS, our findings of impairments in overt judgments also point to a potential role for abnormalities in interoceptive awareness in CS. One possibility that deserves consideration in future research conducted with psychophysiological recordings is that some patients with CS may in fact only have a deficit at the level of interoceptive awareness, rather than the generation of autonomic responses to person cues. Regardless of the outcome of such future research, the present demonstration of overt recognition deficits suggests that CS does not always represent a mirror-image of the deficits in person recognition that are typically present in patients with prosopagnosia (see Behrmann and Plaut, 2013, for review). As such, the present findings also point to a functional organization of person recognition processes in the human mind and brain that may be more dynamic than previously thought, and include behaviorally relevant interactions between covert and overt processes.

## Conflict of Interest Statement

The authors declare that the research was conducted in the absence of any commercial or financial relationships that could be construed as a potential conflict of interest.

## Footnotes

1 http://www.life.com/search

2
